# Novel Materials From the Supramolecular Self-Assembly of Short Helical β^3^-Peptide Foldamers

**DOI:** 10.3389/fchem.2019.00070

**Published:** 2019-02-15

**Authors:** Ketav Kulkarni, Nathan Habila, Mark P. Del Borgo, Marie-Isabel Aguilar

**Affiliations:** Department of Biochemistry and Molecular Biology and Biomedicine Discovery Institute, Monash Univdersity, Melbourne, VIC, Australia

**Keywords:** β-peptide, supramolecular selfassembly, peptide materials, biomaterials, β-amino acid containing peptides

## Abstract

Self-assembly is the spontaneous organization of small components into higher-order structures facilitated by the collective balance of non-covalent interactions. Peptide-based self-assembly systems exploit the ability of peptides to adopt distinct secondary structures and have been used to produce a range of well-defined nanostructures, such as nanotubes, nanofibres, nanoribbons, nanospheres, nanotapes, and nanorods. While most of these systems involve self-assembly of α-peptides, more recently β-peptides have also been reported to undergo supramolecular self-assembly, and have been used to produce materials—such as hydrogels—that are tailored for applications in tissue engineering, cell culture and drug delivery. This review provides an overview of self-assembled peptide nanostructures obtained via the supramolecular self-assembly of short β-peptide foldamers with a specific focus on N-acetyl-β^3^-peptides and their applications as bio- and nanomaterials.

## Introduction

Peptide-based self-assembly is the spontaneous formation of a stable hierarchical structure via a combination of molecular interactions that include hydrogen bonding, hydrophobic interactions, electrostatic interactions, π-π stacking and van der Waals forces (without an external trigger). In general, peptide-based self-assembly is mediated by a variety of secondary structural conformations. Peptides comprised of α-amino acids have the propensity to adopt secondary structures including α-helices, β-sheets and coiled-coils that influence the peptide-based self-assembly processes (Cui et al., 2010; Woolfson and Mahmoud, 2010; Matson and Stupp, 2012; Raymond and Nilsson, 2018). The challenge in the design of novel, functional peptide-based materials is to engineer the constituent peptide monomers that take advantage of these unique secondary structural conformations to establish complementary surfaces that interact non-covalently in a repeating fashion to create the desired nanostructure with favorable mechanical properties.

**Figure 1:**
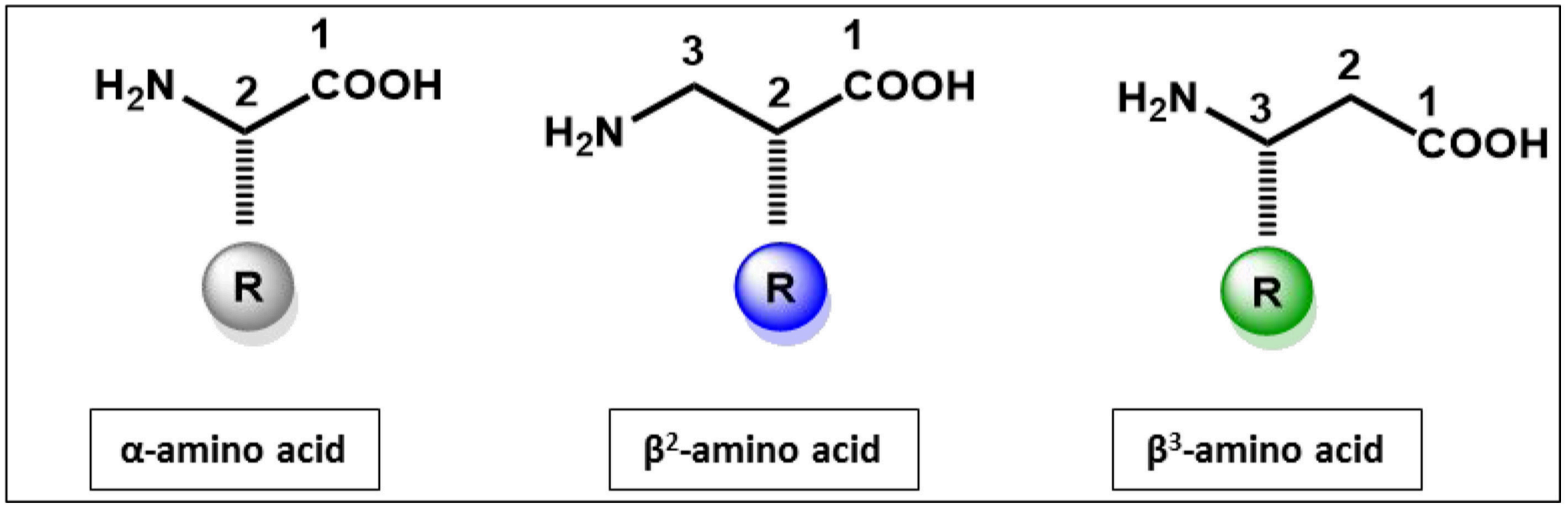
Structural features of α-amino acid, β^2^and β^3^-amino acids.

β-Peptides are polyamides composed of β-amino acids which differ from α-amino acids due to the presence of an extra methylene in the backbone (Cheng et al., 2001; Seebach et al., 2004; Gopalan et al., 2015). The extra CH_2_ group (Figure 1) is inserted between the carboxy terminus and the α-carbon atom. The side chains can be positioned at either the α- or β-carbon, resulting in either β^2^ or β^3^-amino acids.

The incorporation of an extra methylene results in both constitutional and configurational isomers (Steer et al., 2002; Seebach et al., 2004; Aguilar et al., 2007; Clerici et al., 2016) which are either R or S configuration at the α-(C^2^) carbon or the β-(C^3^) carbon, resulting in a total of 4 possible isomers for any given side chain. The advantages of β^3^-peptides over α-peptides depend on the application and whether single substitutions or entire β-peptides are considered. As a peptidomimetic, single substitution of α-amino acids by β^3^- or β^2^-amino acids in α-peptides confers resistance to enzymatic degradation as the extra methylene shifts the scissile bond away from the active site nucleophile, thereby stabilizing an otherwise metabolically sensitive bioactive peptide. β^3^- or β^2^-amino acids may also induce small changes in peptide conformation again leading to different biological activity (Sagan et al., 2003; Cabrele et al., 2014). The majority of studies have utilized β^3^-amino acids in favor of β^2^-amino acids, as the preparation of β^2^-amino acids requires more complex synthetic methods while β^3^ amino acids can be obtained in a 2-step reaction from their α-amino acids counterparts. β^3^-peptides have been used in the design of a wide range of bioactive compounds and peptide and protein mimetics (Fülöp et al., 2006; Aguilar et al., 2007; Checco and Gellman, 2016; Del Borgo et al., 2017; Kiss et al., 2017). These applications range from enzyme inhibitors, receptor agonists and antagonists and inhibitors of protein-protein interactions to antimicrobial agents.

The next major feature of β-peptides is their ability to adopt well-defined helical structures stabilized by hydrogen bonding (Appella et al., 1997, 1999; Seebach et al., 1997, 1998). While there are at least five different helices, including 8-, 10-, 12-, 14-, and 10/12-helix, (Cheng et al., 2001) that have been identified for β-peptides based on the number of atoms in the hydrogen-bonded rings, oligomers of β^3^-peptides are predominantly defined by either a 14- or 12-helical conformation. The 14-helix is stabilized by hydrogen bonding between the main chain amide proton (HN) at position ***i*** and the carbonyl (CO) at position ***i*****+*****2***, forming a 14-membered pattern (Cheng et al., 2001). Similarly, the 12-helix is stabilized by hydrogen bonds between the backbone amides (HN) at positions ***i*** and ***i*** **+** ***3*** (CO). The overall structure of the 14-helix differs from that of the α-helix with a slightly wider radius (14-helix = 2.7 Å and α-helix = 2.2 Å), a lower number of residues per turn (14-helix = 3.0 residues and α-helix = 3.6 residues) and a shorter rise per residue for a given chain length than the α-helix (14-helix = 1.56 Å and α-helix = 1.5 Å). The 12-helix has a radius of 2.3 Å and consists of 2.5 residues per turn with a rise per residue of 2.1 Å (Cheng et al., 2001).

**Figure 2:**
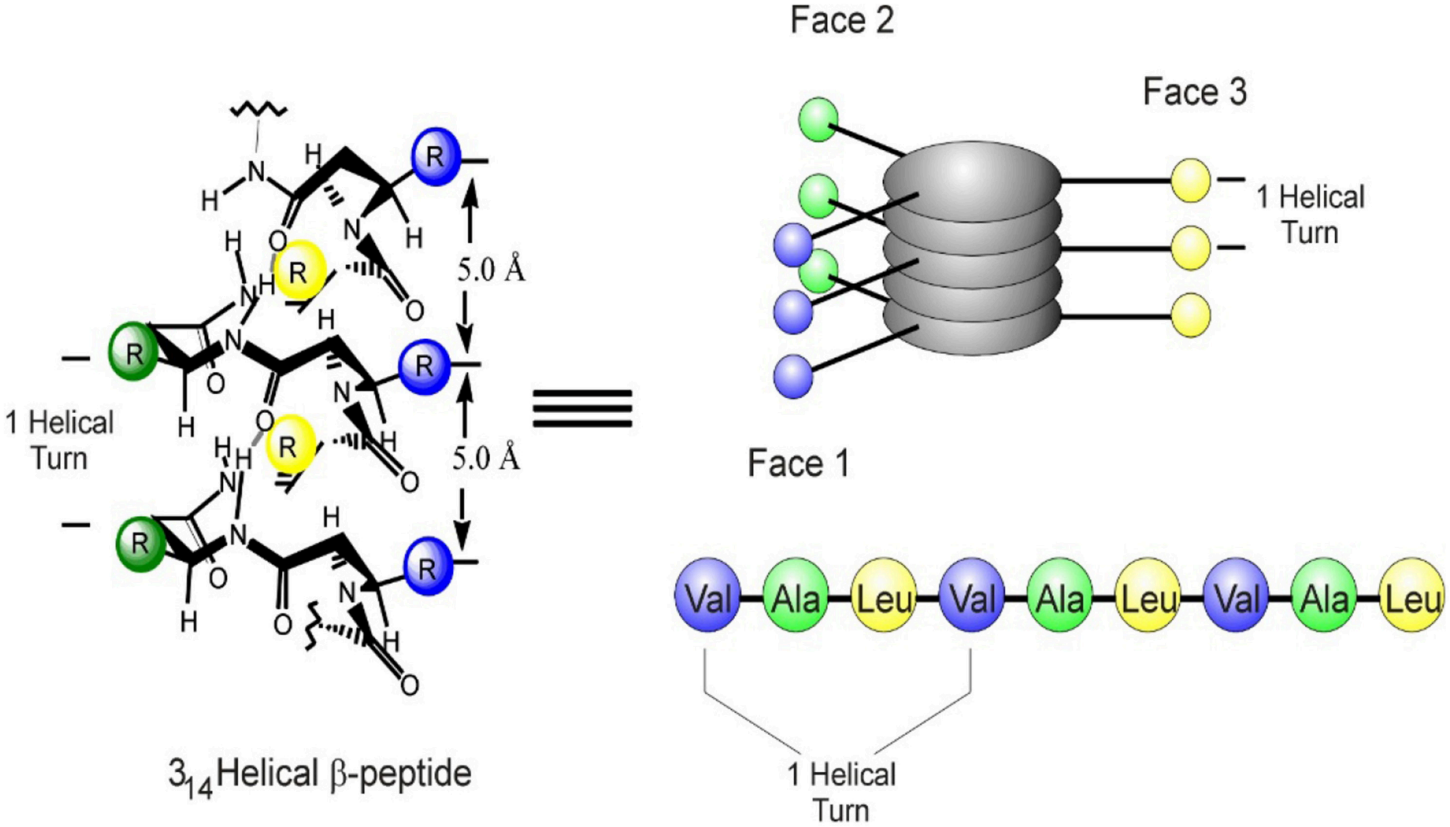
Structure and geometry of a β^3^-peptide 14-helix with the longitudinal alignment of amino acid side chains on the helical surface resulting in a topology that mediates the formation of peptide bundles or for binding to specific targets.

The 14-helix is characterized by ~3 residues per turn, which results in the alignment of the side chains of every fourth residue directly along one face of the helix (Figure 2) (Cheng et al., 2001). These foldamers have been used as a template for a wide range of biomimetic studies as reviewed previously (Martinek and Fulop, 2012; Gopalan et al., 2015; Mándity and Fulop, 2015; Del Borgo et al., 2017). Moreover, the ability of β-peptides and α/β-peptide hybrids to adopt stable secondary structures has also underpinned the design of novel structure-based receptor inhibitors and peptide bundles as protein mimics (Checco et al., 2015a,b; Liu et al., 2018). In this review, we describe how the unique secondary structure of β-peptide fibers has been determined and how this structural template has been exploited to modulate the fiber morphology through lateral interactions, thereby providing new opportunities for the design of novel, stable materials.

## β^3^-Peptide Bundles

Prior to the recent reports of supramolecular self-assembly of β^3^-peptides, a number of groups have demonstrated the assembly of β^3^-peptides into bundles containing 4–10 individual β^3^-peptides (Raguse et al., 2001; Daniels et al., 2007; Goodman et al., 2007, 2008; Giuliano et al., 2009; Wang et al., 2012, 2014; Wang and Schepartz, 2016). A β^3^-peptide bundle arises from the cooperative folding of β^3^-peptides into higher-order quaternary assemblies in solution and exhibit protein-like properties in which the hydrophobic surfaces are buried in the interior core. The initial steps toward creating a specific helical-bundle with β^3^-amino acid oligomers used two oligomers of optically active trans-2-aminocyclohexanecarboxylic acid (ACHC) that assembled into a tetramer and a hexamer. Similarly, a 10-residue β^3^-peptide designed to adopt amphiphilic helical conformation was also observed to self-assemble in aqueous solution to form tetrameric and hexameric bundles (Raguse et al., 2001). This group has also shown the self-assembly of helical quaternary bundles that are arranged in a parallel orientation of oligomers comprising a mixture of α/β-peptides (Raguse et al., 2001; Horne et al., 2007; Giuliano et al., 2009).

**Figure 3:**
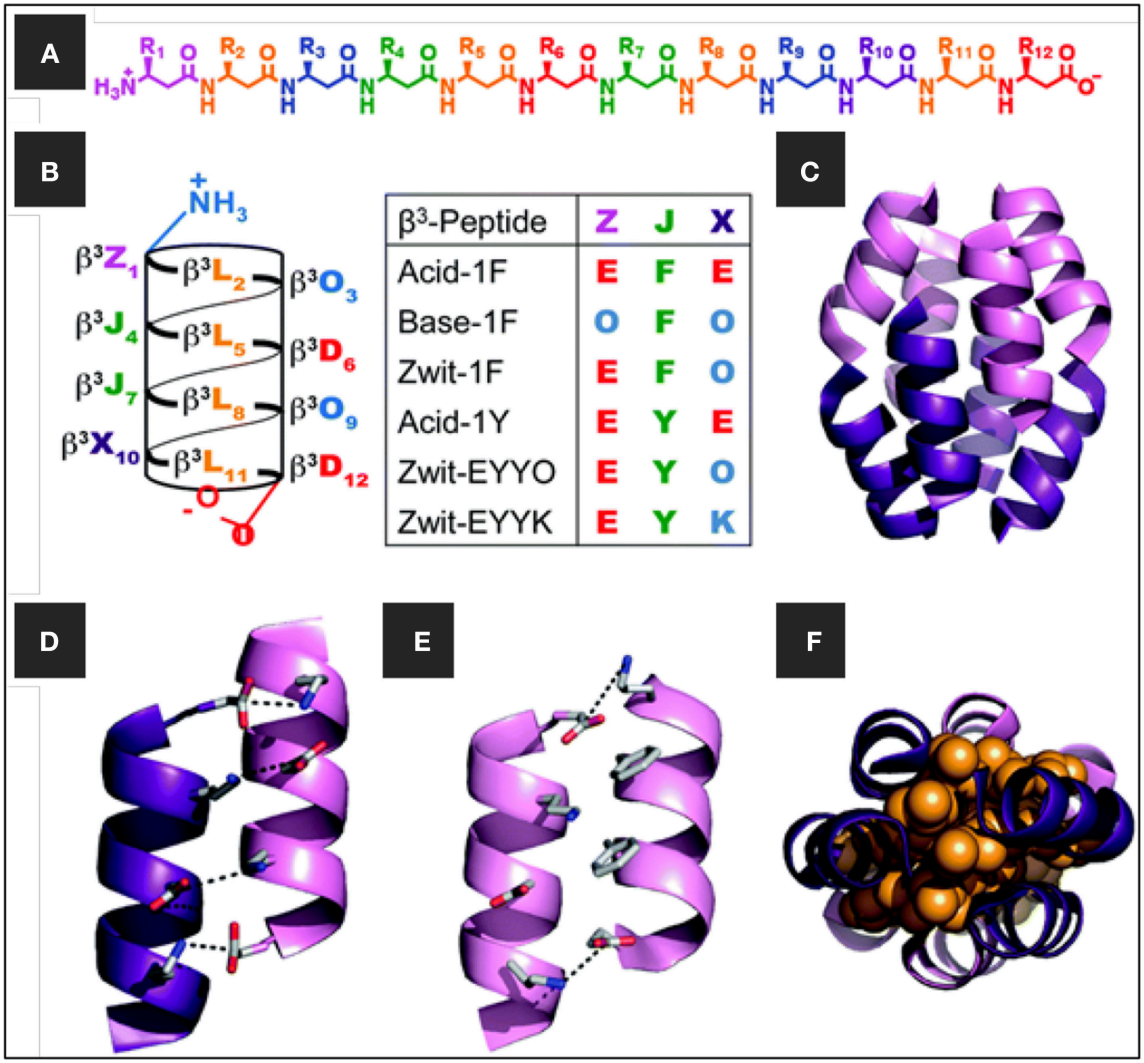
**(A)** Chemical structure of β-dodecapeptide, **(B)** helical net representation of a β-dodecapeptide folded into a 14-helix with the different β^3^-peptide sequences **(C)** β-peptide bundle, the homo-octamer of Zwit-1F, **(D,E)** close-up view of salt-bridging interactions at the **(D)** anti-parallel and **(E)** parallel helical interfaces of Zwit-1F, and **(F)** space-filling model (orange) showing tight packing of β^3^-homoleucine side-chains in the Zwit-1F core. Adapted with permission from Daniels et al. (2007). Copyright 2007 American Chemical Society.

The first stoichiometrically defined octameric β^3^-peptide bundle was designed and characterized with several high-resolution structures (Daniels et al., 2007; Goodman et al., 2007, 2008; Craig et al., 2011). The octameric bundles were made up of β^3^-decapeptides with three distinct faces: a hydrophobic β^3^-Leu face, a salt bridge face of alternating β^3^-Orn and β^3^-Asp residues, and an aromatic surface that contained two β^3^-Tyr or β^3^-Phe residues. The β-peptide oligomer assembled as antiparallel helices with the β^3^-Leu side chains forming the core of the bundle. The same group also described a series of β^3^-peptides which assembled into octameric β-peptide bundles with the aromatic face defined as Acid-1F, Base-1F, Zwit-1F, Acid-1Y, Zwit-EYYO, and Zwit-EYYK (Figure 3). The thermodynamic and kinetic stability of the bundle then allowed this structure to be used as a template for the design and functionalization of new protein-like β^3^-peptide bundles.

As a follow up to this model, the first functional β^3^-peptide helical bundle was recently reported (Wang et al., 2014). The β^3^-peptide bundles were capable of both substrate binding and catalysis of 8-acetoxypyrene-1,3,6-trisulfonate to release the fluorescent product pyranine upon ester hydrolysis. This is useful for optical sensor applications and a fluorescent pH indicator for the physiological range. A combination of kinetic and high-resolution structural analysis suggested the presence of an esterase active site comprising three functional groups, positioned on different strands of the octameric bundle structure.

## Supramolecular Self-Assembly of β^3^-Peptides

### β-Peptides Containing Cyclic β-Amino Acids

**Figure 4:**
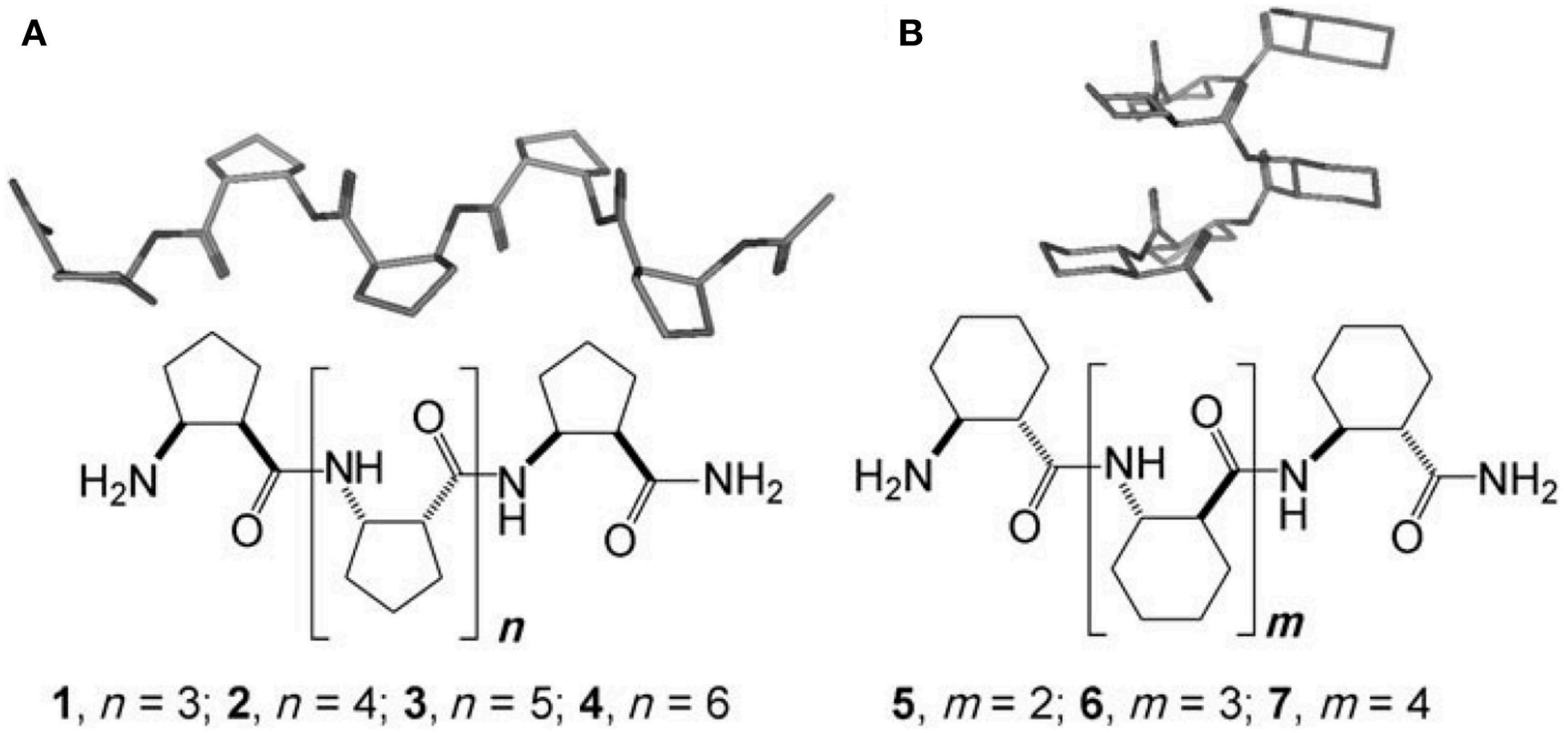
Chemical structures and the secondary structures of **(A)** *cis*-ACPC_n_ (1–4) and **(B)** *trans*-ACHC_m_ (5–7) homooligomers. Reproduced with permission from Martinek et al. (2006). Copyright 2006 John Wiley & Sons.

Supramolecular self-assembly of β^3^-peptides is the formation of a well-defined large structure from the organization of β^3^-peptide monomers. Supramolecular self-assembly of β^3^-peptides can lead to materials ranging from nano- to macroscopic in dimension (Gopalan et al., 2015; Yoo and Lee, 2017). One of the earliest examples of a supramolecular self-assembling β-peptides was composed of either *cis*-(1R,2S)-ACPC_n_ (1–4; ACPC = cis-2-aminocyclopentanecarboxylic acid) or *trans*-(1S,2S)-ACHC_m_ (5–7; ACHC = trans-2-aminocyclohexanecarboxylic acid, which adopt a strand or a helix conformation, respectively, in solution (Figure 4) (Martinek et al., 2006). These β-peptide monomers were shown to undergo supramolecular self-assembly to yield fibrils of pleated-sheet sandwiches and helix-bundle membranes. Another group reported the production of nanostructures from the self-assembly of a cyclobutane β-tetrapeptide (Rúa et al., 2007; Torres et al., 2010). These β-peptides comprising residues derived from (1R,2S)-2-aminocyclobutane-1-carboxylic acid adopted a β-strand-type conformation in solution and modeling indicated that self-assembly was mediated by intramolecular H-bonding to give the sheet structure.

**Figure 5:**
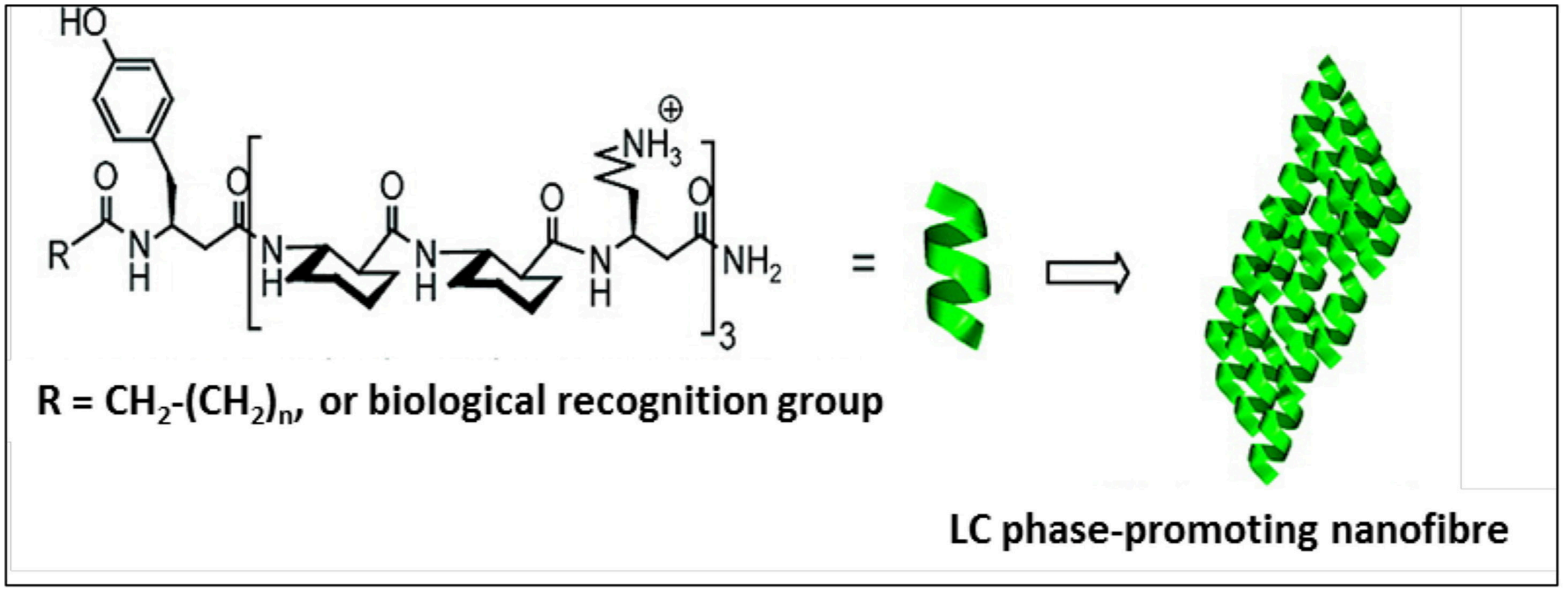
Self-assembled lyotropic liquid crystals with a minimum of three repeats of the ACHC-ACHC-β^3^-Lys triad. Biological recognition group (biotin or the tripeptide RGD) was incorporated at R. Adapted with permission from Pomerantz et al. (2011) Copyright 2011 American Chemical Society.

The self-assembly of β-peptides containing ACHC was also reported by Gellman et al., with the hierarchical organization exhibiting lyotropic liquid crystalline behavior (Pomerantz et al., 2008, 2011). The designed peptide contained a minimum of three repeats of the ACHC-ACHC-β^3^-Lys triad (Figure 5). This peptide folded into a 14 helical conformation, leading to the segregation of the hydrophobic cyclohexyl ring and hydrophilic β^3^-Lys residue. The cyclohexyl units of the neighboring β^3^-peptide interdigitated to form a zipper-like motif—referred to as a “cyclohexyl zipper”—that was achieved by intermolecular association mediated by the amphiphilic nature of the molecule. Structural analysis revealed the existence of two different types of species, globular aggregates, and nanofibers, which were the predominant self-assembled structure that lead to lyotropic LC phase formation.

**Figure 6:**
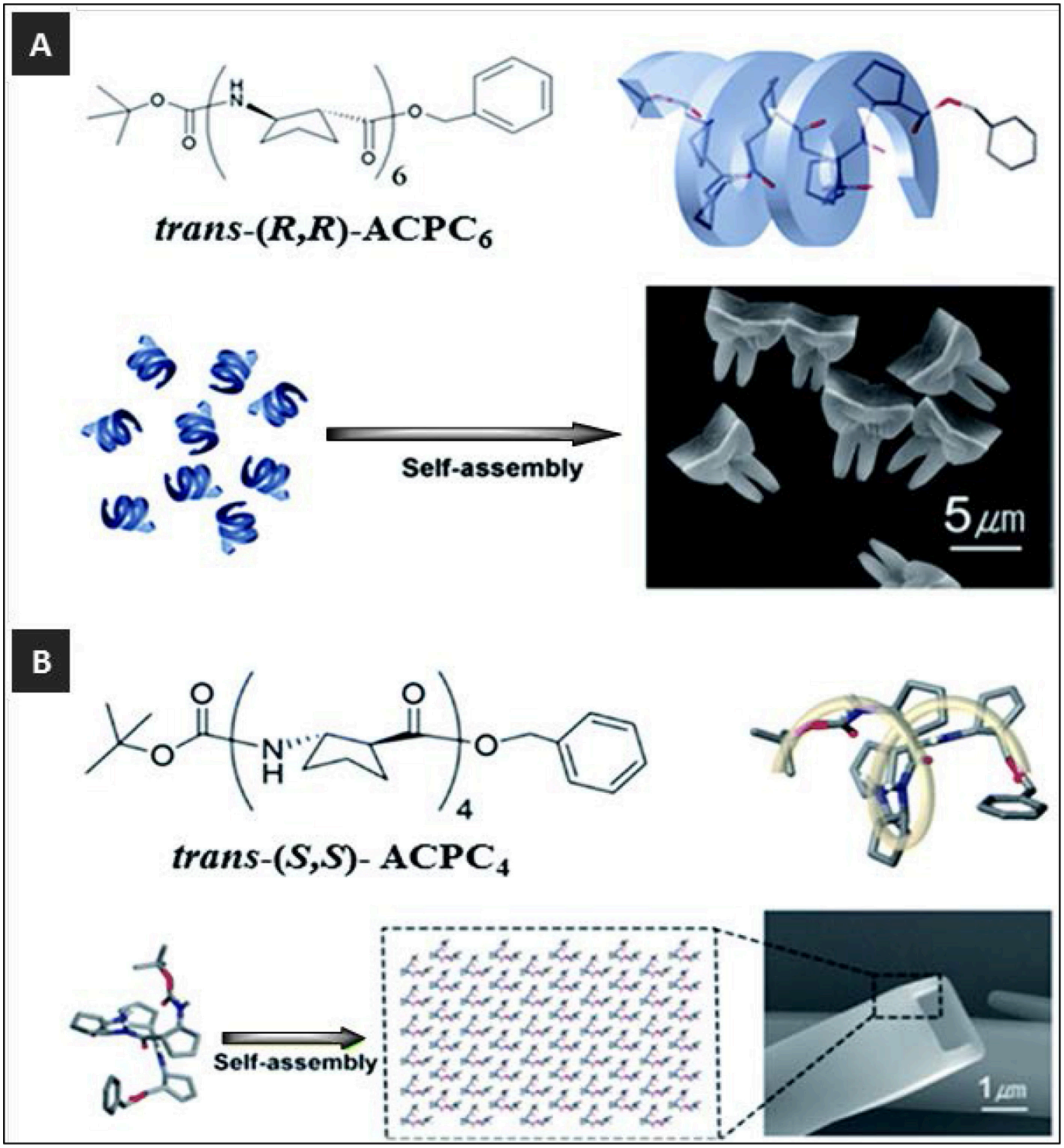
**(A)** Self-assembly of tooth-shaped architectures from trans-(R,R)-ACPC_6_ and **(B)** self-assembly of rectangular microtubes from trans-(S,S)-ACPC_4_. Adapted with permission from Kwon et al. (2011), Copyright 2011 American Chemical Society.

Spontaneous self-assembly of β-peptide monomers comprising of trans-2-aminocyclopentanecarboxylic acid (ACPC) homo-oligomers as a building block have resulted in a number of 3D microstructures (Kwon et al., 2010, 2011, 2015; Kim et al., 2012). These β-peptide monomers—ACPC_6_, ACPC_7_, and ACPC_4_–have been shown to self-assemble into novel 3D tooth-shaped, windmill-shaped and tapered square rod structures and rectangular microtubes (Figure 6) (Kwon et al., 2010, 2011, 2015; Kim et al., 2012; Yoo and Lee, 2017). The monomers adopted a stable right-handed 12-helical conformation in solution. The self-assembly motif is based on the fact that the helix self-assembled in aqueous solution via lateral hydrophobic interactions between the helical faces as well as by head-to-tail intermolecular hydrogen bonding (Kwon et al., 2010). It was concluded that highly ordered anisotropic molecular packing motifs, contained within the foldamer building blocks, are responsible for their unique shapes. The ability of these structures to align and move under a magnetic field at the microscopic as well as macroscopic scales was recently demonstrated by integrating magneto-responsive foldamers within hydrogels (Kwon et al., 2015). These reports highlight the design of biocompatible 3D molecular architectures with different functions and morphologies that could potentially be used for the next generation of biocompatible peptide-based structures.

The approach of substituting β-amino acids to stabilize α-peptides against proteolytic stability has also been applied to the stabilization of α-peptide-based hybrid hydrogels (Yang et al., 2006, 2007). The gelation of these β-amino acid-containing peptide-based hydrogels was triggered by changes in pH and temperature. Another approach utilized mixed α/β-peptides that self-assemble to form hydrogels were shown to be stable to proteolysis and exhibited strong gelation properties (Mangelschots et al., 2016). The self-assembly of N-terminally protected β-alanine-containing dipeptides has also been reported to give a hydrogel which exhibited the controlled release of encapsulated vitamin B derivatives (Nanda and Banerjee, 2012).

### N-Acetyl Acyclic β^3^-Peptide Assemblies

#### Head-to-Tail 3-Point H-Bonding Motif

**Figure 7:**
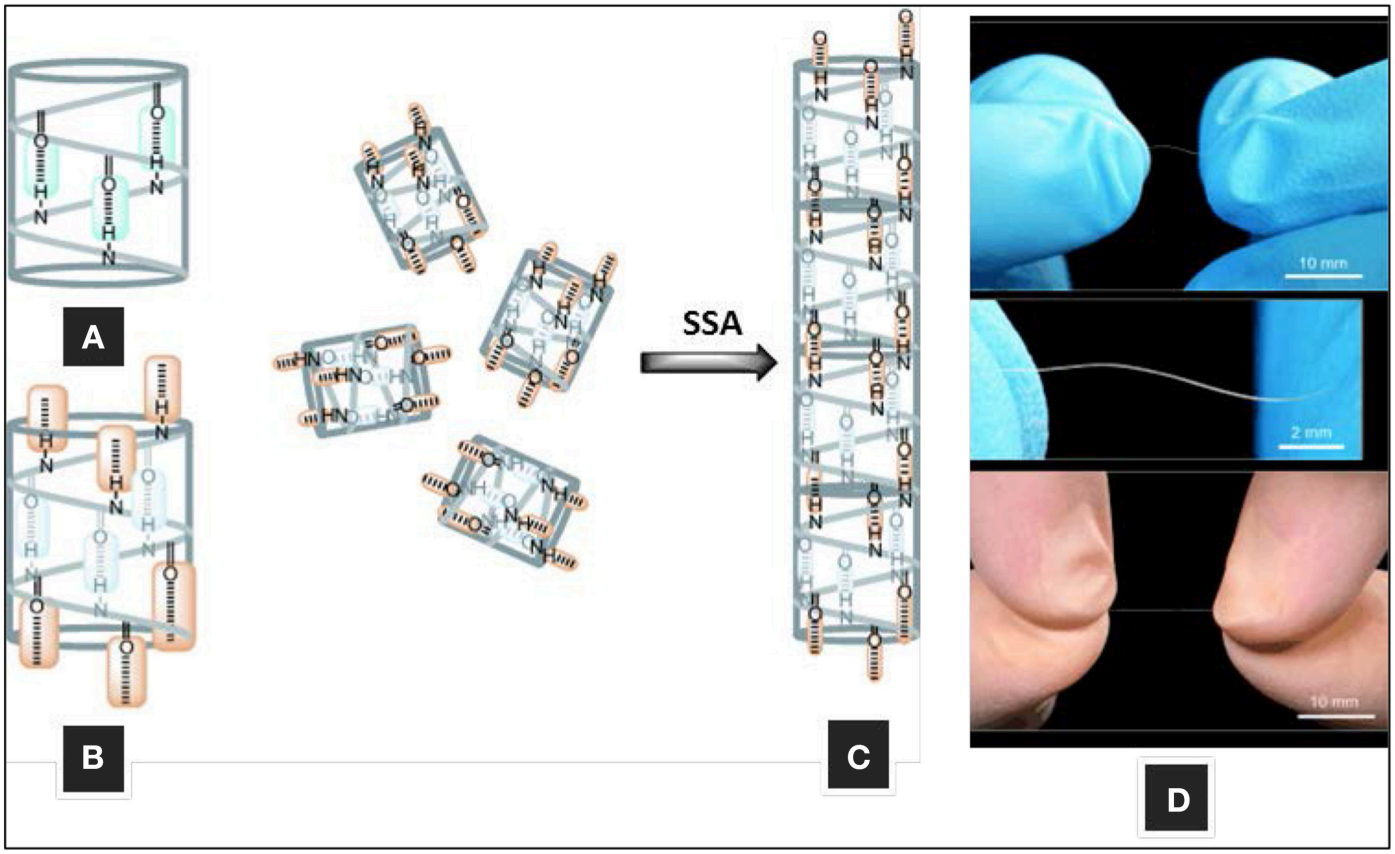
Self-assembly of 14-helical β^3^-peptides. **(A)** Intramolecular hydrogen bonding motif for helix stabilization, **(B)** intermolecular hydrogen-bonding motif **(C)** fiber formation by head-to-tail hydrogen bonding, and **(D)** macroscopic fibers formed by β^3^-hexapeptide A_C_-WKVWEV-OH. Adapted with permission from Del Borgo et al. (2013). Copyright 2013 John Wiley & Sons.

The geometry of the 14-helix means that there are ~3 residues per turn, which results in the alignment of the side chains of every fourth residue directly along one face of the helix (Figure 2) (Cheng et al., 2001; Martinek and Fulop, 2012). This molecular symmetry forms the core of the supramolecular self-assembly of β^3^-peptides and N-acetylation of β^3^-peptides containing at least three β^3^-amino acids and triggers self-assembly via a head-to-tail motif (Del Borgo et al., 2013; Christofferson et al., 2018). X-ray crystallography confirmed that the capping of the N-terminus provides the necessary H-bond donors and acceptors to facilitate a 3-point H-bond motif that drives the axial self-assembly of β-peptides (Del Borgo et al., 2013). The N-acetyl group plays a critical role by providing the third donor-acceptor interaction pair and thus promotes axial self-assembly and fiber growth into higher ordered structures (Figure 7). TEM and atomic force microscopy (AFM) revealed that the peptides with a free N-terminal amine showed no signs of self-assembly, whereas the N-acetylated peptides self-assembled into fibrous morphologies of varying sizes and shapes in different solvents. Interestingly, the structures of two β^3^-tripeptide monomers could be superimposed with a β^3^-hexapeptide to resemble a typical left-handed 14-helical structure with two turns, and internally supported by ***i***, ***i*****+*****2*** intramolecular hydrogen bonds.

The β^3^-peptides self-assembled in water and methanol, forming fibers from several micrometers up to 3 cm in length and ~0.25 mm in diameter within 1 h of incubation (Figure 7). The large fibrillary structures self-assembled from β^3^-peptide monomers comprised of β^3^-amino acids in multiples of three residues. The head-to-tail model of β^3^-peptide self-assembly was shown to be persistent under a variety of conditions with fibers growing into large structures (Del Borgo et al., 2013). The variation in the thickness and morphology of the structures formed indicated that side chain interactions may influence the extent of inter-fibril interactions. The effect of different solvents on the superstructure morphology of a self-assembled β^3^-peptide, Ac-β^3^-[LIA], was thus investigated (Seoudi et al., 2015a). The fibrils interact through a combination of solvophobic, van der Waals and H-bonding interactions, and a change in overall fibril structure was therefore achieved through tuning the relative strengths of the inter-fibril H-bonding, van der Waals and solvophobic interactions. In apolar solvents where the carboxyl-terminal residues do not interact with the solvent, inter-fibril H-bonding drives the formation of assemblies of straight fibers. In contrast, in protic solvents such as alcohols, carboxylic acid residues are solvated, resulting in less prominent inter-fibril H-bonding and a balance between solvophobic and van der Waals effects leads to the formation of dendritic structures. In water, hydrophobic effects dominate over van der Waals interactions, leading to the formation of rope-like twisted structures.

Given the sensitivity of inter-fibril interactions to the solvent environment, the effect of the hydrophobic and steric topography of the 14-helical nanorod on lateral self-assembly was also investigated (Seoudi et al., 2015b). In particular, the fiber structure of three isomeric peptides was studied, comprising the same three β^3^-amino acid residues: β^3^-homoleucine (β^3^-L), β^3^-homoisoleucine (β^3^-I) β^3^-homoalanine (β^3^-A) to give peptides Ac-β^3^- [LIA], Ac-β^3^- [IAL], and Ac-β^3^- [ALI]. AFM imaging revealed different superstructures for each peptide, confirming that the surface topography of different non-polar side chains can drive an effective approach for the generation of different self-assembled nanomaterials. These studies also demonstrated that the combined effect of the relative spatial distribution of different non-polar side chains and the solvent polarity have an important role in the self-assembly of these compounds and the resulting self-assembled nanomaterials.

The majority of N-acetylated β^3^-peptides which have been reported contained either three or six β^3^-amino acids, i.e., multiples of three residues, resulting in one or two complete turns of the 14-helix, respectively. Therefore, to investigate the effect of perturbing the helical turn on the β^3^ peptide self-assembly, β^3^-tetrapeptides (Ac-β^3^-[ALIA], Ac-β^3^-[SLIA], and Ac-β^3^-[KLIE]) with 1 1/3 turns of the helix were also studied (Seoudi et al., 2016). All peptides underwent self-assembly confirming that the head-to-tail H-bonding motif was unaffected by the sequence length and composition. Moreover, side chain polarity did not affect the ability of β^3^-tetrapeptides to self-assemble into helical nanorods.

**Figure 8:**
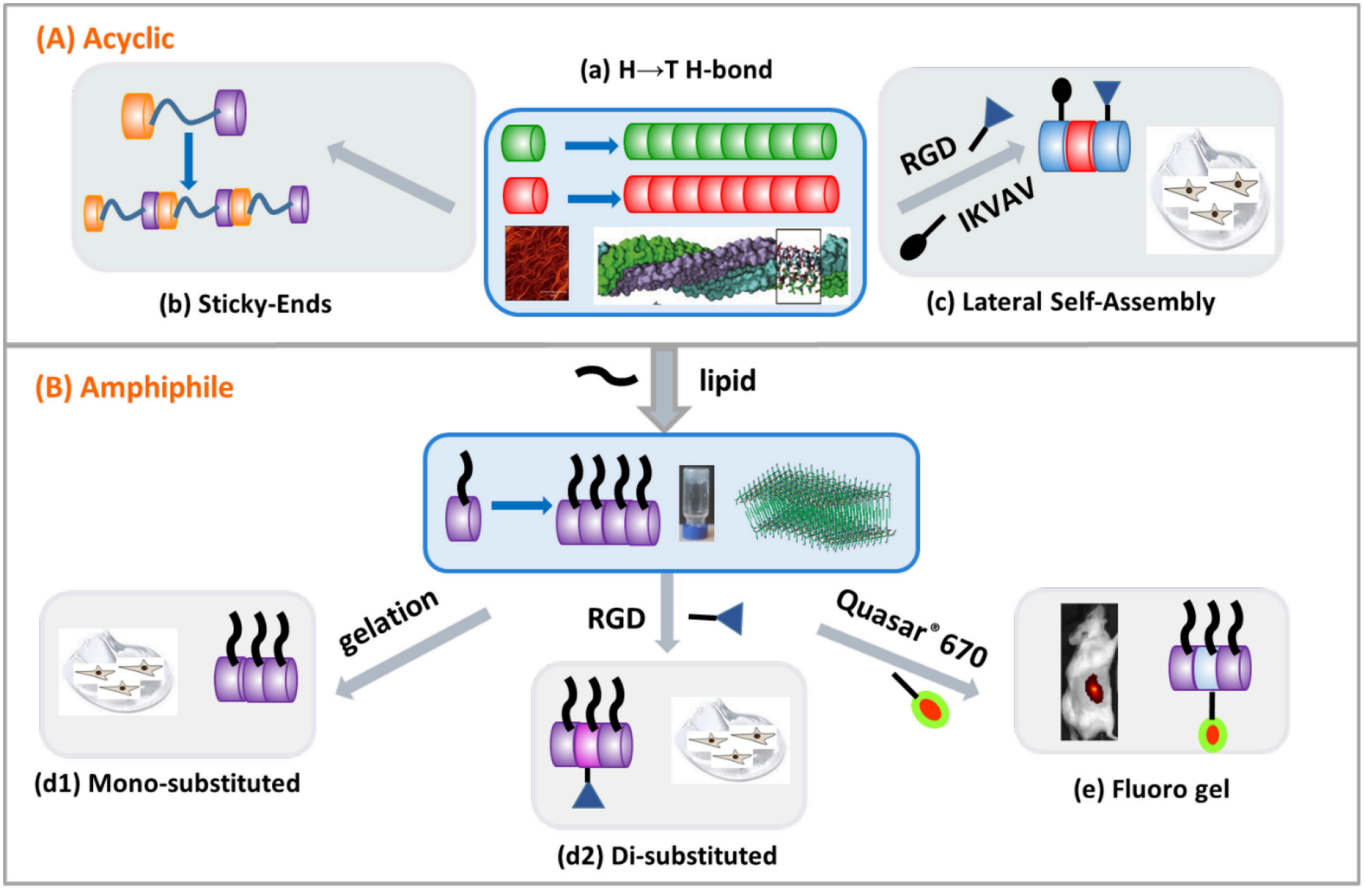
Modular design of β^3^-peptide biomaterials by exploiting the head-to-tail (H → T H-bond) 3-point H-bonding motif. Monomers comprised of **(A)** acyclic β^3^-amino acids or **(B)** lipo- β^3^-amino acids. **(A) (a)** Template self-assembly motif, **(b)** sticky ends, and **(c)** controlled morphology through lateral interactions. **(B) (d1)** mono-substituted hydrogel, **(d2)** disubstituted hydrogel and **(e)** fluoro hydrogel.

A structural model for a three-stranded helical coiled coil derived from a self-assembled N-acetyl-β^3^-peptide was recently reported using X-ray fiber diffraction of Ac-β^3^-[LIA] and molecular dynamics simulations (Christofferson et al., 2018). The triple 14-helical coiled coil motif was characterized by a hydrophobic core with the twist along the fiber axis arising from interactions between the β^3^-homoleucines and β^3^-homoalanines of adjacent tripeptides on the solvent-exposed surface of the fiber. The computational methods established in this study can now be used to determine the fibril structure for other materials derived from N-acetylated-β^3^-peptides and allow rational engineering of nano- and biomaterials. The modular platform for the design of novel biomaterials has now been established, as shown schematically in Figure 8A and discussed in the sections below.

#### β^3^-Tripeptides Act as Sticky Ends

While the 14-helical structure of β^3^-peptides has been shown to be essential for the H-bonding required for self-assembly, the strength of the 3-point H-bonded self-assembly motif was investigated by integrating a rigid or flexible linker between two β^3^-tripeptide monomers to create a hybrid β^3^-peptide scaffold (Del Borgo et al., 2018). The flanking β^3^-peptides were comprised of three β^3^-amino acids and the results showed that incorporation of either a flexible hexyl linker or a rigid phenyl linker did not perturb self-assembly, demonstrating the dominance of the 3-point H-bonded motif in β^3^-peptide self-assembly (Figure 8A–b). This ability of the β^3^-tripeptides to act as sticky ends during self-assembly was further tested by incorporation of the tripeptide cell-adhesion epitope, α-arginylglycylaspartic acid (RGD) resulting in an α/β hybrid peptide. This peptide also self-assembled to give fibers which acted as bioscaffolds for 2D culture of primary hippocampal neurons, further expanding the design template for new materials based on β^3^-peptides.

#### Control of Morphology Through Lateral Interactions

As a consequence of the 14-helical self-assembly of β^3^-peptide monomers, the side chains are aligned laterally, resulting in a high degree of symmetry along the periphery of the β^3^-peptide nanorods. This provides an opportunity to functionalize the structure at the side chain position without affecting the self-assembly. Most significantly, in contrast to other peptide-based self-assembling materials, N-acetylated β^3^-tripeptides form fibrous scaffolds in solution or on surfaces, irrespective of the amino acid sequence or solution conditions (Del Borgo et al., 2013; Gopalan et al., 2015). In order to characterize the impact of larger side chains on the self-assembly and to generate a biological scaffold, β^3^-tripeptides were functionalized with anchoring cell-adhesion signals IKVAV or RGD (Figure 8A–c) (Luder et al., 2016). Both epitopes were incorporated into a β^3^-peptide monomer as a functionalized side chain by reducing the γ-azido-β^3^-homoalanine (β^**3**^-Az) side chain using triphenylphosphine, followed by solid phase peptide synthesis (SPPS). The integration of α-peptide epitopes into the β^3^-peptide sequence did not disrupt their ability to self-assemble.

High-resolution analysis by AFM and EM of the fibrous structures generated by the IKVAV-containing peptide showed a helical periodicity of ~5 nm in height. If the diameter of a β^3^-tripeptide 14 helix is 0.5 nm and the length of IKVAV side chain in an extended structure is ~2 nm, the overall diameter of the peptide monomer is estimated to be 2.5 nm, indicating that the observed fibrils are commensurate with the two peptide nanorods. Most significantly however, the size of the IKVAV side chain did not hinder peptide self-assembly via the head-to-tail motif. Moreover, the fibrils exhibited uniform width, further indicating that the incorporation of a large hydrophobic side chain inhibited lateral assembly. The central role of the head-to-tail H-bond motif in the self-assembly process was further demonstrated whereby mixtures of IKVAV and RGD functionalized β^3^-peptides were shown to self-assemble to give heterogeneous fiber scaffolding.

To determine whether the IKVAV and RGD motifs were accessible and able to support cell adhesion, the ability of mouse primary cardiac fibroblasts and bone marrow-derived macrophages (BMDM) to adhere to the β^3^-tripeptide scaffolds composed of either homogeneous or mixtures of both cell-adhesion motifs was investigated. While there was no additive effect on the growth of cardiac fibroblasts with any of the scaffolds, fibroblasts grown on scaffolds comprised solely of peptide with an RGD side chain showed a significant decrease in cell viability, reflecting the over-stimulation of cells by excess RGD, an effect that was reversed by reducing the amount of RGD peptide in the mixture. In comparison, a different effect of the scaffolds was observed for the BMDMs. While the RGD-peptide did not affect cell viability, mixtures of RGD and IKVAV peptides significantly were shown to be biocompatible and resulted in greater cell adherence compared to controls. This demonstrated the ability to tailor the scaffolds for particular cell types with an ability to present a variety of cell-specific ligands attached to β^3^-peptide monomers to support and modulate bioactivity of cells (Luder et al., 2016).

#### β^3^-Peptide Amphiphiles

Peptide amphiphile is a term used to describe a molecule that comprises a hydrophobic lipid chain coupled to a hydrophilic oligopeptide sequence (Cui et al., 2010; Matson and Stupp, 2012; Dehsorkhi et al., 2014; Webber et al., 2016). Peptide amphiphiles self-assemble into a range of nanostructures including nanofibers (Hartgerink et al., 2002; Niece et al., 2003), nanotubes (Cui et al., 2010), twisted and helical ribbons (Pashuck and Stupp, 2010), micelles (Trent et al., 2011) and nanotapes (Miravet et al., 2013). The most widely studied class of peptide amphiphiles consists of one alkyl tail that is attached to the N-terminus which can self-assemble in water and through the influence of pH (Dehsorkhi et al., 2013), light (Muraoka et al., 2009), temperature (Hamley et al., 2013), proteolysis (Webber et al., 2011) and ionic strength (Dehsorkhi et al., 2014), with chemical diversity that is well tolerated within the new nanostructure (Paramonov et al., 2006; Cui et al., 2010; Stupp, 2010; Stupp et al., 2013; Korevaar et al., 2014).

Given that self-assembly of β^3^-peptides is unperturbed by the steric bulk of the side chain while the presence of a hydrophobic sequence further inhibits the lateral assembly resulting in uniform fiber morphology, β^3^-peptide amphiphiles in which a lipid chain moiety is incorporated into a β^**3**^-peptide template have been synthesized and shown to undergo self-assembly. The first study reporting a β^**3**^-peptide-based amphiphile described the synthesis of a β^**3**^-amino acid containing two acyl chains which, when mixed with 1-palmitoyl-2-oleoyl-sn-glycero-3-phosphocholine, self-assembled to give lipid-like vesicles (Capone et al., 2008). More recently, an N-acetylated β^**3**^-peptide containing a lipid chain was synthesized, which self-assembled to form a hydrogel which was both a mechanically stable and biocompatible hydrogel (Motamed et al., 2016), illustrated in Figure 8B–d1. The tri-β^**3**^-peptide Ac-β^**3**^-[AzKA] was synthesized containing a β^**3**^-homolysine (β^**3**^-K) residue to enhance peptide solubility in aqueous buffer. The C14 alkyl chain was then introduced on solid support by reducing the azide moiety on β^**3**^-Az using triphenylphosphine followed by acylation with myristic acid to form the final peptide structure. This C14 acylated tri-β^**3**^-peptide resulted in the formation of fibers with uniform diameter and self-assembled into a fibrous mesh retaining water (Figure 8B–d2). The resulting hydrogel was shown to be non-toxic, exhibited a stiffness compatible with brain tissue, and supported the adherence and proliferation of dopaminergic neurons. Given that the alkyl chain was important for facile hydrogel formation, the design of the β^**3**^-peptide amphiphile was further extended by synthesizing a functional β^**3**^-peptide amphiphile by incorporating a C14 acyl chain and the cell adhesion motif RGD (Kulkarni et al., 2016). This was achieved by first synthesizing a new β^3^-amino acid with an allyloxycarbamate (alloc-) protected aminoethyl amide side chain to allow for the orthogonal attachment of functionalities to the β^3^-tripeptide amphiphile using solid-phase peptide synthesis (Kulkarni et al., 2016). The peptide acylated tri-β^**3**^-peptide when co-dissolved with C14 and formed a stable hydrogel at a concentration of 10 mg mL^−1^. The dual-functionalized β^3^-tripeptide showed enhanced L929 cell (mouse fibroblastic cell line) adhesion by increasing the RGD concentration from 2 to 8% (Kulkarni et al., 2016) (Figure 8B–d2). These materials were proteolytically stable, were shear thinning and supported the growth of fibroblasts, thereby further demonstrating the potential of β^3^-peptide-based materials in tissue regeneration.

#### Fluoro-β^3^-Peptides for *in vivo* Imaging

The spontaneous self-assembly of N-acetylated β^3^-peptides irrespective of sequence and side chain structure, combined with the ability to introduce desirable properties by appending the β^3^-peptide monomers with functional payloads, opens the possibility to produce materials with different chemical and optical properties. As a result, the ability to introduce a fluorescent moiety in β^3^-peptide-based hydrogels for *in vivo* imaging applications has been recently demonstrated (Kulkarni et al., 2018). The incorporation of fluorescent peptide monomers within the fiber gel is necessary to maintain hydrogel fluorescence and to monitor the fate of the material *in vivo*. To generate a fluorescent β^3^-tripeptide amphiphile, Quasar® 670, a far-red emitting dye with excitation at 644 nm and emission at 670 nm, was attached to a lipidated β^3^-tripeptide. Super-resolution imaging through stimulated emission depletion (STED) microscopy confirmed that the fluorescent monomer successfully co-assembled with a C14 lipidated β^3^-tripeptide, with the fiber matrix showing even distribution of the fluorophore. Interestingly, the fluorescent β^3^-peptide did not co-assemble successfully within a matrix containing β^3^-peptide, confirming the important role of the lipid side chain in directing hydrogel formation. Rheological measurements showed the incorporation of the fluorescent peptide had no effect on hydrogel stiffness and was comparable to that of ECM found subcutaneously (Figure 8B–e). Following administration of the fluorescent hydrogels to mice via subcutaneous injection, visualization of the gel implants using real-time *in vivo* animal imaging showed no degradation 14 days post-implantation. Overall, these results demonstrate that β^3^-tripeptide fluorescent hydrogels which can be easily detected *in vivo* and functionalized with different bioactive epitopes may provide a template for the design of stable implantable materials for *in vivo* biomedical applications.

## Conclusions and Outlook

The supramolecular self-assembly of β^3^-peptide foldamers is now generating a new class of nanomaterials by exploiting the persistent formation of the β^3^-peptide 14-helical template to achieve a new level of molecular engineering. Depending on the application, peptide-based self-assembled systems exhibit several advantages over other organic and inorganic self-assembled systems in terms of biocompatibility, low toxicity and potential for functionalization. More recently, the self-assembly of β^3^-peptides has led to novel nanomaterials that have significantly expanded the potential applications of β^3^-peptide foldamers (Gopalan et al., 2015; Del Borgo et al., 2017). We recently reported a novel mode of self-assembly of N-acetylated β^3^-peptides which leads to the formation of a wide range of nanomaterials (Figure 8). In particular, these β^3^-peptides comprised of different acylic amino acid sequences, self-assemble into fibers of different dimensions in aqueous or organic solvents. Moreover, X-ray crystal and fiber diffraction studies have further confirmed the formation of a 14-helical structure which allows the formation of a unique 3-point H-bonding motif where the N-acetyl group contributes to the 3-point H-bonding interactions.

Significantly, the head-to-tail H-bonding motif dominates the self-assembly irrespective of the order and number of residues within the β^3^-peptide sequence, to the extent that the sequence can be disrupted through the introduction of rigid or flexible linkers or new functionalities that can be introduced on different sections of the tri-β^3^ peptide while not impeding the self-assembly. This sequence independent “head-to-tail” self-assembly model, in combination with the unique 14-helical structure of the β^3^-peptides monomer, readily permits the decoration of the monomer with specific biorecognition motifs without impacting on their ability to form fibers. A consequence of this shape-persistent self-assembly motif is the ability to exploit this template to modulate morphology through lateral side-chain interactions. This offers the opportunity for the introduction of a wide variety of functions by modifying the side chains of the β^3^-amino acids, without perturbation of the self-assembly motif. Our understanding of the intricacies observed in the different β^3^-peptide self-assembled morphologies, in terms of molecular interactions, highlights the crucial interplay between various non-covalent interactions, the sensitivity of inter-fibril interactions to the solvent environment and the effect of the hydrophobic and steric topography of the β3-peptide 14-helix on lateral self-assembly. This has led to the control over the morphology through lateral interactions, allowing for the first time the creation of 2D and 3D functional β^3^-peptide materials. The inherent flexibility in this unique design, as well as the ability to functionalize and tailor it for different applications, provides a new strategy for the development of novel bio- and nanomaterials via N-acetyl β^3^-peptide supramolecular self-assembly.

## Author Contributions

KK, NH, MD, and M-IA contributed conception and design of the study. KK and NH wrote the first draft of the manuscript. All authors contributed to manuscript revision, read, and approved the submitted version.

### Conflict of Interest Statement

The authors declare that the research was conducted in the absence of any commercial or financial relationships that could be construed as a potential conflict of interest.
